# Food insecurity and hospital resource utilization

**DOI:** 10.1002/jhm.70362

**Published:** 2026-06-10

**Authors:** Adolfo L. Molina, Ariel Carpenter, Mary Orr, Samantha L. Hanna, Dana Woodruff, Cynthia Deerman, Cassi Smola, Erin E. Shaughnessy, Chang L. Wu

**Affiliations:** ^1^ Department of Pediatrics, University of Alabama at Birmingham Children's of Alabama Hospital Birmingham Alabama USA; ^2^ Division of Social Work Children's of Alabama Hospital Birmingham Alabama USA

## Abstract

**Background:**

Food insecurity (FI) is an important pediatric social risk factor associated with worse health outcomes. However, there is a paucity of data on FI's effects on hospitalized children and hospital‐based reutilization.

**Methods:**

This single‐center, retrospective cohort study included children admitted between 2020 and 2023 to evaluate the effect of FI on hospital utilization (length of stay [LOS], emergency department [ED] revisits, and readmission). FI was identified using the two‐question Hunger Vital Sign^TM^ at admission, with patients categorized as food secure, food insecure, or missed/refused screening. Multivariable generalized estimating equations, utilizing binomial and negative binomial distributions, were employed to calculated adjusted odds ratios (aOR) and incidence rate ratios (aIRR), respectively, while accounting for patient‐level clustering. Models were adjusted for age, sex, race, ethnicity, language, and insurance type.

**Results:**

We analyzed 31,553 pediatric hospitalizations in the analysis. Results demonstrated that patients in the missed/refused screening group had a significantly increased LOS, with a 73% increase in expected hospital duration (aIRR: 1.73; 95% confidence interval [CI]: (1.60–1.89) *p* < .0001) compared with the food‐secure group. In contrast, no significant associations were found between documented FI and LOS, ED revisit, and 30‐day readmission after adjusting for patient clustering.

**Conclusions:**

While the relationship between FI and utilization is multifactorial, these findings suggest that patients who are missed by or refuse screening represent a high‐acuity cohort with significantly higher resource utilization. Addressing social risk during hospitalization remains an important opportunity for resource connection, and the high utilization among the unscreened population highlights a critical area for improving screening equity and clinical outreach.

## INTRODUCTION

Food insecurity (FI) is defined as the limited or uncertain availability of nutritionally adequate and safe foods, or the ability to acquire acceptable foods in socially acceptable ways.^1^ FI represents an important pediatric social risk factor given the critical role of adequate nutrition in supporting normal growth and development.^2^ Screening for FI is essential in pediatrics to promote food security for all children.^3^ Research, primarily undertaken in primary care and emergency department (ED) settings, demonstrates detrimental associations of FI on chronic health, including asthma, obesity, diabetes, overall poor health, and even mental health.^4^, ^5^, ^6^, ^7^, ^8^

There is growing literature to support the need for social determinants of health (SDoH) screening in the inpatient setting, including evidence demonstrating a relationship between SDoH risk factors and increased hospital utilization.^9^, ^10^ However, many of these studies rely on broad geographic indicators, which may not accurately capture individual risk.^11^, ^12^ Comprehensive SDoH screening can be challenging to implement in routine healthcare use. FI screening with the Hunger Vital Sign^TM^ offers the advantage of being a brief, two‐question tool with the potential to provide a clear actionable response, both for acute assistance as well as longitudinal management.^13^ Further, evidence suggests that providers may feel more comfortable with asking about FI in comparison to other SDoH categories.^14^ However, there is a paucity of data on FI's effects on hospitalized children and hospital‐based reutilization.^15^ Our study leverages a large, single‐center cohort to examine how FI, identified through inpatient screening, impacts subsequent ED revisits and rehospitalizations.

## METHODS

In this single‐center, retrospective cohort study, we assessed hospital resource utilization among admitted children who were identified as experiencing household FI. All patients admitted from August 2020 through March 2023 to our free‐standing, quaternary‐care children's hospital were included. Household FI was defined as a positive screen on The Hunger Vital Sign (HVS)™, a two‐question assessment that has been previously shown in the ambulatory setting to successfully screen for FI.^10^ The primary predictor was categorized as not food insecure, food insecure, and missed screen/refused study participation. Primary outcomes were hospital length of stay (LOS) measured in days, ED return visit, and hospital readmission (all‐cause, 30‐day, and 365‐day), and mortality. Covariates included standard demographics: age, sex, race, ethnicity, primary language, and insurance type.

Our hospital's goal is to screen all admitted patients for FI, regardless of admitting diagnosis or service of admission. Screening was completed by bedside nursing staff, and the assessment was incorporated in the hospital's standard intake screening questionnaire. A positive screen constituted an answer of either “Sometimes true” or “Often true” to either of two questions assessing household concerns about food running out or not lasting due to financial constraints answered by either the patient or caregiver. While every attempt was made to screen patients at the first opportunity, some situations required screening to be deferred until later in the hospitalization (e.g., a critically ill patient arriving in the intensive care unit [ICU] in the middle of the night). Families were permitted to refuse participation. Notably, our institution has an established pathway for patients and their families identified as food insecure. Families identified to have FI are provided food during hospitalization, a box of food at discharge, a discussion of women, infants and children or supplemental nutrition assistance program benefits applications, and local food bank resources facilitated through an automated social work consult.

### Statistical analysis

Descriptive statistics summarized the study cohort using means with standard deviations for normally distributed continuous variables, medians with interquartile ranges (IQR) for non‐normally distributed data, and frequencies with proportions for categorical variables. To account for the dependency and clustering of multiple hospitalization encounters contributed by individual patients, we utilized generalized estimating equations (GEEs) with empirical (sandwich) standard error estimates. An exchangeable correlation structure was specified a priori as the primary approach to account for within‐patient clustering; however, in instances of model nonconvergence, an independent correlation structure was utilized as a robust alternative.

For binary outcomes (ED returns, hospital readmissions, and mortality), multivariable GEE models were specified with a Binomial distribution and Logit link to estimate adjusted odds ratios (aOR). For length of stay (LOS), a Negative Binomial distribution with a Log link was employed to calculate adjusted incidence rate ratios (aIRRs), accounting for the characteristic right‐skew and overdispersion of healthcare utilization data. Covariates were clinically selected a priori and included age, sex, race, ethnicity, primary language, and insurance type. Notably, race and ethnicity were included as fixed effects to adjust for structural and systemic inequities rather than biological determinants.

Potential multicollinearity was rigorously assessed using Cramer's *V* derived from *χ* ^2^ tests of independence for all pairwise combinations of categorical variables. Particular scrutiny was applied to the intersection of race, ethnicity, and language; however, no pairwise associations exceeded our predefined threshold (*V* > 0.60), and subsequent sensitivity analyses confirmed model robustness. All analyses were conducted using SAS software, version 9.4 (SAS Institute Inc.).

This retrospective analysis was reviewed by our institutional review board (IRB) and deemed not to meet the criteria for human subjects research.

## RESULTS

We included 31,553 hospitalizations from 21,630 patients in the analysis cohort (Table 1). Patients had a median age of 6.3 years, were mostly male (54.2%), White (60.6%), non‐Hispanic/Latino (92.7%), English‐speaking (94.3%), and had government insurance (79.8%). A majority (81.7%) of patients were screened, and 2.6% (824 patients) were identified as food insecure. In bivariate analysis (Table 2), FI was significantly associated with increased 365‐day ED revisit (*p* < .001). In multivariable analysis, FI remained associated with increased 365‐day ED revisit (aOR: 1.21, *p* < .05). After accounting for clustering of repeated hospitalizations within patients, the associations between FI and 365‐day outcomes were no longer statistically significant. There was no significant association detected between FI and LOS, rehospitalization, or mortality (rare in all groups <0.05%) (Table 3).

**Table 1:** Demographics of study population.

Characteristic	Complete study cohort, *n* (%)	Not food insecure, *n* (row %)	Food insecure, *n* (row %)	Missed/refused, *n* (row %)
	31553 (100)	24945 (79.06)	824 (2.61)	5784 (18.33)
Age				
0–2 years	9342 (29.61)	6943 (27.83)	215 (26.09)	2184 (37.76)
3–5 years	6116 (19.38)	4990 (20.00)	163 (19.78)	963 (16.65)
6–12 years	7616 (24.14)	6199 (24.85)	215 (26.09)	1202 (20.78)
13+ years	8479 (26.87)	6813 (27.31)	231 (28.03)	1435 (24.81)
Sex				
Male	17095 (54.20)	13480 (54.06)	468 (56.80)	3147 (54.42)
Female	14448 (45.80)	11456 (45.94)	356 (43.20)	2636 (45.58)
Race				
White	19125 (60.61)	15442 (61.90)	416 (50.49)	3267 (56.48)
Black	11489 (36.41)	8796 (35.26)	377 (45.79)	2316 (40.04)
Other	939 (2.98)	707 (2.83)	31 (3.76)	201 (3.48)
Ethnicity				
Non‐Hispanic	29248 (92.69)	23217 (93.07)	689 (83.62)	5342 (92.36)
Hispanic	2305 (7.31)	1728 (6.93)	135 (16.38)	442 (7.64)
Primary Language				
Non‐English	1790 (5.67)	1309 (5.25)	125 (15.17)	356 (6.15)
English	29763 (94.33)	23636 (94.75)	699 (84.83)	5428 (93.85)
Insurance				
Government	25189 (79.83)	19727 (79.08)	764 (92.72)	4698 (81.22)
Private	6007 (19.04)	4956 (19.87)	46 (5.58)	1005 (17.38)
Other	357 (1.13)	262 (1.05)	14 (1.70)	81 (1.40)

**Table 2:** Bivariate comparison of food insecurity and hospital outcomes.

	Not food insecure, *n*(%) 24945 (79.06)	Food insecure, *n*(%) 824 (2.61)	Missed/refused, *n*(%) 5784 (18.33)	*p V*alue
ED readmit (30 days)	1553 (6.23)	66 (8.01)	350 (6.05)	*p* = .0920
ED readmit (365 days)	5896 (23.64)	232 (28.16)	1340 (23.17)	*p* = .0067
Rehospitalization (30 days)	3144 (12.60)	110 (13.35)	727 (12.57)	*p* = .8118
Rehospitalization (365 days)	7274 (29.16)	257 (31.19)	1864 (32.23)	*p* < .0001

	**Not food insecure** median (IQR)	**Food insecure** median (IQR)	**Missed/refused** median (IQR)	*p* Value
Length of stay (days)	2.00 (1–5)	2.00 (1–6)	3.00 (1–7)	*p* < .0001

Abbreviations: ED, emergency department; IQR, interquartile range.

**Table 3:** Food‐insecurity and hospital resource outcomes (respondents “not food insecure“ are referenced).

	Unadjusted analysis		Adjusted for covariates		Adjusted for covariates and clustering	
	Food insecure odds ratio, *n* (95% CI)	Missed/refused odds ratio, *n* (95% CI)	Food insecure adjusted odds ratio, *n* (95%CI)	Missed/refused adjusted odds ratio, *n* (95% CI)	Food insecure adjusted odds ratio, *n* (95% CI)	Missed/refused adjusted odds ratio, *n* (95% CI)
ED readmit (30 days)	1.31 (1.01–1.70)*	0.97 (0.86–1.09)	1.28 (0.99–1.65)	0.92 (0.82–1.04)	1.13 (0.75–1.70)	0.93 (0.80–1.09)
ED readmit (365 days)	1.27 (1.08–1.48)*	0.97 (0.91–1.04)	1.21 (1.03–1.41)*	0.92 (0.86–0.98)*	1.19 (0.97–1.46)	0.91 (0.84–0.98)*
Rehospitalization (30 days)	1.07 (0.87–1.31)	1.00 (0.91–1.09)	1.03 (0.84–1.27)	1.01 (0.92–1.10)	1.03 (0.76–1.38)	1.01 (0.91–1.11)
Rehospitalization (365 days)	1.10 (0.95–1.28)	1.16 (1.09–1.23)*	1.04 (0.89–1.21)*	1.15 (1.09–1.23)*	1.03 (0.84–1.25)	1.16 (1.08–1.24)*

*Note*: Adjusted for age, sex, race, ethnicity, language, and insurance.

Abbreviations: CI, confidence interval; ED, emergency department.

* *italics p* < .05.

Patients in the Missed/Refused category represented 18.3% of the cohort. This group showed distinct utilization patterns compared with the other two groups. Specifically, children who were either missed in screening/refused participation had a 73% increase in hospital days (*p* < .0001). In contrast, those who were food insecure had a 10.6% increase in stays that was not statistically significant. In adjusted analysis, the Missed/Refused group had significantly higher odds of 365‐day rehospitalizations (aOR: 1.16, 95% CI: 1.08–1.24) but lower odds of 365‐day ED revisits (aOR: 0.91, 95% CI: 0.84–0.98).

## DISCUSSION

Our study revealed notable differences in outcomes for patients who were “Missed/Refused” FI screening, demonstrating an unexpected statistically significant pattern of decreased adjusted odds of 365‐day ED revisits and increased adjusted odds of 365‐day rehospitalizations, as well as a 73% longer length of stay. This “missingness” potentially serves as a proxy for high clinical acuity or social complexity. In the acute setting, families of children with higher severity of illness may be less available for SDoH screenings due to the crisis state of the admission or the frequency of medical interventions.

Furthermore, the increased utilization in this group suggests that the most vulnerable populations may be systematically missed by current screening workflows. While documented FI did not independently predict utilization in the final model, the high resource consumption of the unscreened population indicates that “missing” screening data are not random. These findings are challenging to interpret and highlight the importance of understanding operational barriers to universal screening to ensure high‐risk populations are not systematically excluded from screening. Future efforts should focus on optimizing screening equity, ensuring that children with the longest hospitalizations and highest acuity are not overlooked in social risk assessments.

Initially, our multivariable models suggested that FI was associated with an increased risk of readmission across the overall patient population. However, after accounting for clustering of repeated hospitalizations within individual patients, this association was no longer significant. This finding indicated that the initially observed risk was largely driven by a small, highly vulnerable subset of children who experienced multiple hospitalizations. Thus, the relationship between FI and rehospitalization likely reflects multifactorial and unmeasured structural and systemic barriers that limit certain families' ability to engage with hospital screening and follow‐up processes, rather than FI alone. It is also possible that our hospital's provision of short‐term food support for families identified as FI may have temporarily mitigated the impact of FI on 30‐day utilization outcomes to some extent.

Despite the lack of relationship to utilization outcomes, identification of FI to promote food security is especially important in pediatrics due to the importance of growth and development.^3^ Hospitalization represents a unique opportunity to address pediatric FI at a time when children's hospitals can actively address FI by providing meals during hospitalization and other resources at discharge. Research has also shown that actively addressing social needs is associated with improved health.^16^ Additionally, the American Academy of Pediatrics recommends FI screening, and the two‐item Hunger Vital Sign^TM^ screen is an easy SDoH screen to be integrated into care, given its brevity.^3^, ^13^ This approach aligns with evidence that systematic screening and referral can increase families' use of community resources.^17^

### Limitations

Our study was limited in that it found an underestimation of FI, with only 2.6% of the total population and 3.4% of those screened endorsing FI, as compared with state‐wide estimates of 20%.^18^ The small proportion of patients identified as FI also limits the interpretability of these results and may underestimate the true prevalence and impact of FI in this population. While our estimate of FI in the present study is similar to previous nationally identified rates of FI in a hospital setting, this potential under‐identification may lead to the introduction of selection bias and possible underestimation of the effects of FI.^19^ It is unclear what led to this large divergence in estimated versus identified FI, but it has been noted in prior studies.^20^ We hypothesize multiple potential reasons, including: timing of questions at the point of hospital admission/arrival to the floor (when caregivers may be experiencing higher stress and thus less likely to disclose FI), nursing discomfort with asking the questions, and stigma against positive responses.^21^ Prior studies have shown significant improvements in screening through written questionnaires rather than verbal assessment by a healthcare provider.^22^, ^23^ Other studies have shown that using nonclinical staff to conduct FI screening can improve FI disclosure, likely by reducing social desirability bias.^22^, ^24^ We hope in the future to leverage our electronic health record to enable families, both before outpatient check‐in as well as during inpatient stays, to self‐screen for FI privately with an electronic device without direct questioning, in order to limit the stigma associated with verbal questioning.

Additionally, approximately 18% of patients were not screened during this voluntary, nurse‐led period highlighting real‐world barriers to FI screening. Our institution has since moved to mandatory screening through institutional acceptance of the importance of FI screening, and we have shown improved overall screening rates. This experience highlights that, albeit a brief screen, successful implementation requires institutional commitment and supportive workflows. Furthermore, the Hunger Vital Sign^TM^ has been validated in an outpatient setting and not in a hospital setting. Dewitt et al. and Lee et al. also note in their study the unique distinction between household FI, assessed in our study, and that of the transient phenomenon of inpatient FI brought on by the stressors exclusive to a hospitalization, which we did not assess.^25^, ^26^ We were unable to include ICU admission as portions of our dataset originated from a legacy electronic health record that is no longer accessible, and thus, we were unable to include ICU admission in our model, limiting our ability to assess the contribution of illness severity to missed screening. Finally, our inability to distinguish between missed or refused screening may obscure important differences between these subgroups, potentially limiting the interpretability of the seemingly paradoxical utilization outcomes for this category.

## CONCLUSION

In this retrospective cohort study, patients not screened for FI demonstrated higher health resource utilization. The observed associations between FI and hospital utilization did not reveal significance after accounting for clustering of repeated hospitalizations within patients, suggesting a more complex relationship between FI and our hospital utilization metrics. Universal screening for FI remains an important effort to identify families at risk of unmet basic needs and provides opportunities for timely intervention during a hospitalization. Future research targeting best practice screening strategies to limit stigma and improve screening are also needed to accurately identify patients and families in need in order to better understand the complex relationship of FI with healthcare utilization.

## CONFLICT OF INTEREST STATEMENT

The authors declare no conflict of interest.

## Data Availability

The data that support the findings of this study are available on request from the corresponding author. The data are not publicly available due to privacy or ethical restrictions.
